# Treating Behavioral Addictions With Augmented Reality and Virtual Reality: Scoping Review

**DOI:** 10.2196/77011

**Published:** 2026-04-30

**Authors:** Felicia Xin Rou Chiew, Genevieve Huimin Li, Victrine Tseung, Jing Shi

**Affiliations:** 1 Occupational Therapy National University Health System Singapore Singapore; 2 Occupational Therapy Tan Tock Seng Hospital Singapore Singapore; 3 Brain and Heart Nexus Research Program University of Ottawa Heart Institute Ottawa, ON Canada; 4 Health and Social Sciences Singapore Institute of Technology Singapore Singapore

**Keywords:** behavioral addictions, virtual reality, augmented reality, intervention, video gaming, gambling, scoping review, behavioral harms, treatment, cue exposure

## Abstract

**Background:**

The use of augmented reality (AR) and virtual reality (VR) to address addictive behaviors such as substance use disorders and gambling disorders has been growing. However, little has been done to explore the use of AR and VR in the treatment of other behavioral addictions.

**Objective:**

This scoping review aims to provide an overview of existing literature on AR and VR interventions for behavioral addictions. Specifically, the research questions are as follows: (1) What behavioral addictions or behavioral harms are being treated using AR and/or VR? (2) What AR and/or VR treatment interventions are being used to treat these behavioral addictions?

**Methods:**

This scoping review was conducted based on the framework first proposed by Arksey and O’Malley, later refined by Levac et al, and further outlined in the Joanna Briggs Institute (JBI) Manual for Evidence. The literature was searched in the following databases: CINAHL, PsycArticles, PsycInfo, PubMed, and Web of Science, with Google advanced search complementing the search on Feb 22, 2023. Studies were screened by 2 independent reviewers based on inclusion criteria (all ages; any behavioral addiction, problematic behavior, or behavioral harm; AR or VR treatments and interventions) and exclusion criteria (pornography, sexual, and paraphilic disorders). Discrepancies were resolved by third and fourth reviewers. As this study is a scoping review, risk of bias was not assessed. Data were extracted and presented in tabular form as well as through conceptual analysis as a narrative summary.

**Results:**

A total of 9 studies were included in this review, 4 studies on video gaming and 5 studies on gambling behaviors. Participants’ age ranged from 12 to 65 years. Only the use of VR was identified. VR was used as a platform for cue exposure therapy and skills training in both gaming and gambling disorders. VR therapy was effective alone or in combination with other treatments and was comparable to traditional interventions. No adverse effect was reported in the studies.

**Conclusions:**

VR is efficacious in treating behavioral addictions and can replace or be used in conjunction with traditional methods. Future directions include using VR with other psychotherapy or relapse prevention, applying VR to treat other addictions, and investigating harmful side effects of VR use. The frequency and duration of sessions can also be optimized. A limitation of this study is that there may be other documents beyond those published and searched in gray literature that could not be included in this review due to time and resource restrictions. The use of AR in the treatment of behavioral addictions did not yield any results in this review. However, VR application in behavioral addiction is promising, potentially efficacious, and capable of multiple applications.

**International Registered Report Identifier (IRRID):**

RR2-10.4309/EGCN4808

## Introduction

### Background

Behavioral addictions are characterized by repetitive and rewarding behaviors that disrupt an individual’s life [1]. Only 2 behavioral “addictions”—gambling disorder and internet gaming disorder—are acknowledged in the *ICD-11* (*International Classification of Diseases, 11th Revision*) [1]. Substance use disorders and compulsive sexual behavior disorders are excluded, as they are classified separately under the *ICD-11* due to their distinct clinical profiles. Other behavioral harms such as exercise-related harms [2], mobile phone harms [3], and social media harms [4] are also widely debated. Systematic reviews and meta-analyses indicate that the worldwide prevalence of gaming disorders is approximately 3.05% [5], while the prevalence of gambling disorder is approximately 1.29% [6]. These findings show similarities to the prevalence of certain substance use disorders, estimated at 2.6% [7]. The World Health Organization (WHO) presentations on addictions at international forums in 2017 and 2019 [8] highlight an increase in attention on behavioral addictions.

Both recognized behavioral addictions have been linked to negative consequences. Internet gaming disorders have been associated with issues such as lower academic achievement, sleep loss [9], difficulties with interpersonal relationships, and effects on career opportunities [10]. While there exist studies that highlight potential benefits of gaming, especially for open-world games [11,12], this study focuses on excessive or problematic gaming behaviors that extend beyond healthy recreational use. These patterns, when persistent, may lead to significant psychosocial and physical difficulties. Gambling disorders may negatively impact personal relationships [13], employment, fitness, sleep [14], and overall health and well-being [15]. Recently, there has been a significant rise in the development of preventative strategies, assessment tools, and treatment methods for gaming and gambling disorders. Other potentially problematic behaviors that are less studied, such as social media use, shopping [16], exercise [17], work [18], general internet use [19], and other behaviors that do not involve substance intake, may also negatively impact psychosocial and physical well-being.

Current treatments like cognitive behavioral therapy (CBT) have proven effective in lessening problematic behaviors by empowering clients with motivational and cognitive tools to initiate change and avoid relapse [20]. Additionally, CBT helps individuals develop skills for regulating their emotions and problem-solving [20]. Other common interventions include mindfulness-based cognitive therapy to help with managing cravings [21] and family therapy and support groups to provide social support for patients [22].

### Technological Advances in Treatment Modalities

Mental health interventions have recently welcomed new innovations and experiences that showcase new technologies [23]. Two of these technologies are augmented reality (AR) and virtual reality (VR). AR technology establishes a computer-generated setting that produces a unique reality where real and virtual elements are perceived simultaneously and interactively [24]. One therapeutic application of AR technology is an AR-powered smart training program designed to teach tooth brushing and enhance oral hygiene for individuals with intellectual disabilities [25]. As a more advanced technology, VR uses computer graphics, motion sensors, and 3D display technologies that fully immerse users [26]. VR technology has shown positive outcomes in enhancing functional performance among adults in psychosocial rehabilitation [27]. Until recent years, VR faced limitations due to its high cost and poor quality of graphics and content available. However, growing interest in VR from the video game industry has made VR more accessible by reducing costs and increasing capabilities, resulting in an increasing uptake of VR technology [28].

The use of AR and VR to address mental health issues such as anxiety, substance use disorders, and gambling disorders has also been growing. AR and VR have been used in exposure therapy, where positive results were shown. One study found that an AR exposure app with graded tasks, varying in intensity and duration, effectively reduced the participants’ subjective fear and disgust toward spiders [29]. Another study found that VR therapy effectively decreased craving and improved self-control in individuals with substance use disorders by exposing them to stimulating cues, ranging from pictures of items to immersive environments [30]. In addition, VR has proven helpful in the treatment of anxiety through its ability to generate virtual environments that induce anxiety, serving as a form of exposure therapy. Conducting exposure therapy in a virtual setting instead of in real life lowers the risk of worsening symptoms that might occur with direct exposure, making it a safer approach [31]. VR has also been used in providing role-playing opportunities for vocational training for people diagnosed with schizophrenia. Participants who received a 10-hour virtual job interview training had higher blind-rated scores by human resource members and higher self-rated confidence levels [32]. While there is growing interest in AR and VR as treatment modalities, research exploring the use of AR and VR in the treatment of behavioral addictions has not been consolidated. Hence, this scoping review aims to provide a comprehensive overview of existing literature on the use of AR and VR treatment for behavioral addictions or behavioral harms and to synthesize knowledge gaps for future research.

## Methods

### Overview

A scoping review was performed systematically according to the methodological framework proposed and augmented by Arksey and O’Malley [33], Levac et al [34], and the Joanna Briggs Institute (JBI) Manual for Evidence Synthesis [35]. The scoping review framework has 6 stages as described in the subsequent sections. The protocol for this study was first submitted for publication on January 27, 2023, and published after peer review [36].

### Stage 1: Identifying Research Questions

This scoping review aims to provide a comprehensive overview of existing literature on the use of AR and VR treatment for behavioral addictions or behavioral harms and synthesize knowledge gaps for future research. Our review questions were as follows:

1. What behavioral addictions or behavioral harms are being treated using AR and/or VR?
2. What AR and/or VR treatment interventions are being used to treat behavioral addictions?

### Stage 2: Identifying Relevant Studies

A comprehensive search was conducted in 5 databases, including CINAHL, PsycArticles, PsycInfo, PubMed, and Web of Science. Additionally, gray literature and Google advanced search were searched (the first 10 pages were screened). The search strategy aimed to identify studies published in English in the last 10 years where full-text publications were available. This was to account for the validity of recent technological advancements and to include a larger number of relevant articles that can be used in this scoping review.

To identify relevant literature, the selected databases above were searched using keywords after consultation with an academic librarian (see protocol [36] for a full list of keywords used). Boolean operators were applied to combine search terms logically. “OR” was used to group synonyms, ensuring the inclusion of all relevant terms. “AND” was used to combine the main keywords, such as “behavioral addiction,” “virtual reality,” and “intervention,” so that only studies addressing all 3 areas were retrieved. Quotation marks were placed around multiword phrases to search for exact matches, while truncation symbols were used to capture variations in word endings. New keywords found in identified articles were included in the final searches across all databases. The full list of search terms used can be found in Multimedia Appendix 1. Finally, reference lists of all articles and reports were hand-searched for additional studies to ensure comprehensive identification of literature [35].

### Stage 3: Study Selection

#### Inclusion and Exclusion Criteria

Studies were selected based on the inclusion and exclusion criteria to meet the scope of the inquiry. The literature included in this review consists of published articles in peer-reviewed journals using all types of methodologies, including protocols. Gray literature is also included to minimize publication bias through Google advanced search. Only literature published in English was screened, reflecting the language expertise of the reviewers; nevertheless, sources of evidence from any country were eligible for inclusion. Inclusion criteria for participants were studies that involved people who exhibit behavioral addictions at all ages. Concepts of AR and VR interventions that can be used in treating behavioral addictions were included, such as assessments, perspectives, and outcomes measured for these interventions. Exclusion criteria were reviews and conference abstracts. Research that studied pornography addiction and compulsive sexual behaviors was excluded due to separate categorizations of compulsive sexual behavior disorder and paraphilic disorders from the *ICD-11* [1].

#### Study Identification

The study selection consisted first of screening titles and abstracts according to the inclusion and exclusion criteria by 2 independent reviewers (FXRC and GHL). If titles and abstracts matched the inclusion and exclusion criteria of the scoping review, a full-text review commenced. Two reviewers (FXRC and GHL) worked independently to review and extract relevant articles before importing them into Zotero (Corporation for Digital Scholarship), an open-source reference management software that eliminated duplicates of citations retrieved from multiple databases before screening [37]. A third and fourth reviewer (JS and VT) were consulted for ambiguities until a consensus was reached. Reasons for the exclusion of full-text studies that did not meet the inclusion criteria were recorded and reported in this scoping review. The search and screening processes were presented in a PRISMA-ScR (Preferred Reporting Items for Systematic Reviews and Meta-Analyses extension for Scoping Reviews) flow diagram [38].

### Stage 4: Charting the Data

Data were extracted from selected articles and recorded in a data extraction table in accordance with the methodology of scoping reviews [35]. The data extraction table was used to collate the extracted data based on identified data fields. These data fields include type of behavioral addiction, aim of study, treatment details, control groups, outcome measures, results, future directions, and conclusion. The first 2 authors (FXRC and GHL) independently extracted data from all the full texts. The last 2 authors (JS and VT) then cross-checked all the extractions.

### Stage 5: Collating, Summarizing, and Reporting the Results

Results are described and collated according to the PRISMA-ScR checklist [38] and the JBI methodological guide for scoping reviews [35] (Multimedia Appendix 2). A conceptual analysis was then carried out to identify and synthesize the key findings emerging from the included studies into main themes. Common characteristics of included studies were identified, and then comparisons were made between these studies under each identified concept. These were presented as a narrative summary.

### Stage 6: Consultation Exercise

The sixth stage is an optional consultation stage of scoping reviews [33]. The purpose of this stage is to gather additional insights, perspectives, or references from stakeholders. Furthermore, this stage assesses whether findings resonate with real-world experiences to enhance the relevance and applicability of this scoping review. For this review, we reached out to 6 stakeholders—identified based on the research team’s established professional networks—including academics, digital developers, and clinicians, about our findings on the identified preliminary literature findings from stage 5. A set of questions, along with a draft of this scoping review manuscript, was emailed to identified stakeholders and consultants for their feedback.

## Results

### Search Results

**Figure 1:**
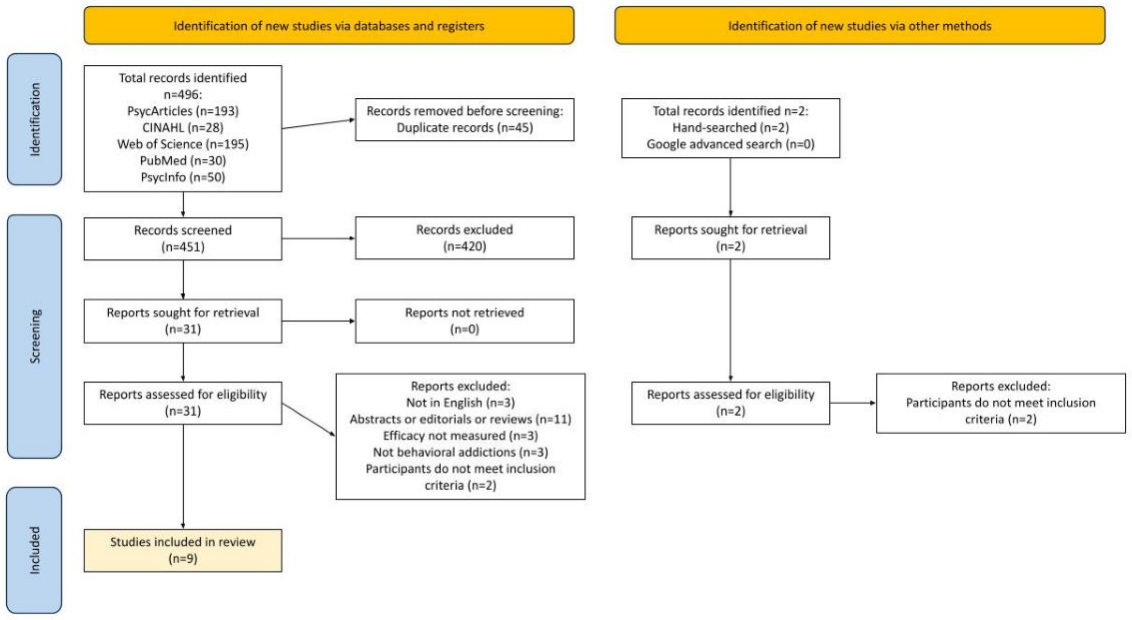
PRISMA (Preferred Reporting Items for Systematic Reviews and Meta-Analyses) flowchart.

Five databases were searched on February 2, 2023. An overview of the data identification and selection process can be found in the PRISMA (Preferred Reporting Items for Systematic Reviews and Meta-Analyses) flowchart (Figure 1). A total of 496 studies were retrieved, of which 45 were duplicates and 420 did not meet the inclusion criteria. In total, 31 full-text studies were assessed for eligibility, which resulted in 9 studies [39-47] for inclusion in this scoping review.

### Study Characteristics

Four studies [43-46] were on video gaming, while 5 studies [39-42,47] explored gambling behaviors. All 9 studies explored the use of VR as an intervention, while no studies used AR. The reviewed articles were from Canada (n=3) [39,41,47], Turkey (n=1) [46], Italy (n=1) [40], and South Korea (n=4) [42-45]. Among the studies that provided information on the age range (n=5) [39,40,44,46,47], they covered ages from 12 to 65 years. Studies on gambling behaviors [39-42,47] recruited participants aged 18-65 years, while studies on gaming behaviors [43-46] recruited younger participants aged 12-25 years. Furthermore, when it came to the sex of the participants recruited, 5 of the studies [42-46] exclusively recruited male participants. One study [47] recruited participants who were mainly White, while another [45] carried out its study on Asian participants. The other 7 studies [39-44,46] did not state the ethnicity of participants. Overall, participants across these studies represented a wide age range and were predominantly male. Among the 9 studies, 6 [39,41-43,45,47] were experimental designs, 2 [44,46] were randomized controlled trials, while one [40] was a study protocol for an experimental design. The characteristics and research aims of the 9 articles are presented in the extraction table titled, “Summary of virtual reality applications in behavioral addictions” (Multimedia Appendix 3).

Four themes were identified in the included studies: (1) the application of VR is predominantly for reducing urges, (2) VR therapies are generally efficacious compared to other treatments, (3) adverse effects are not found in the varied uses of VR treatment for behavioral addictions, and (4) further research is needed to support the use of VR in behavioral addictions treatment.

### Applications of VR Are Predominantly for Reducing Urges

Across all 9 studies, VR was used either in the treatment of gambling (n=5) or gaming disorders (n=4). No other types of behavioral harms were found. The application of VR varied across the studies. VR was mostly used as a platform for cue exposure therapy (CET) [39-44]. CET is a behavioristic psychological approach where individuals are deliberately exposed to relevant addictive cues with the aim of extinguishing conditioned responses associated with the cue by systematically pairing them with a lack of the addictive substance or behavior [48,49]. When CET is conducted through VR, users are immersed in an environment where they are exposed to activity-related environmental stimuli. Giroux et al [41] used a virtual environment featuring 5 video lottery terminals along with other gambling-associated stimuli, including people who are gambling on video lottery terminals, a billiard table, and alcoholic beverages. Similarly, Park et al [42] included gambling-related cues such as navigating and playing a casino game, discussing gambling, and witnessing a jackpot scene. Park et al [43] exposed participants to gaming cues that they frequently play in video games, such as scenes of battling, leveling up, or shooting. Whereas Shin et al [44] exposed participants to scenes of video game invitations, the internet café entrance, and conversations on gaming.

VR was also used as a medium to teach specific skills, such as refusal skills and skills on managing game-related family conflicts. This was done by adopting VR as an environment for participants to engage in a role-playing situation to practice the aforementioned skills [44,45]. Shin et al [44] had participants reject gaming invitations through practicing refusal skills. These participants were told to refuse the avatars’ invitations to play together in a game. In contrast, Shin et al [45] targeted resolving and minimizing game-related conflict with family through risk-benefit analysis and perspective-taking exercises. In the risk-benefit analysis, participants were asked to consider the benefits and disadvantages of halting the game immediately and to evaluate the impact of each example on them using a visual analog scale embedded in the VR program. In the perspective-taking exercise, they were asked to voice out the parents’ behavior from their own perspective, then think about it from the parents’ perspective. They were subsequently tasked to rate the extent to which they were able to understand the parents’ perspective using the same visual analog scale. VR offers a realistic setting by which to practice real-life scenarios using different avatars and visual environments.

One study adopted VR to play sports games to study the therapeutic effects of VR therapy and aerobic training on fitness, physical activity, and anxiety in those with gaming disorder [46]. The VR therapy group played different sports games, including boxing, hurdles, and beach volleyball, on an Xbox Kinect 360 (Microsoft Corp). The games involved simultaneous body movements of the participants, including jumping and hitting movements. This helped to encourage more physical activity and prevent the worsening of gaming disorder [46]. The last study used VR to evaluate the effect of gambling goals on chasing behavior [47]. Chasing behavior refers to the persistent pursuit of losses through continued gambling, involving a pattern of escalating wagers and increased risk-taking in an effort to recoup money lost during previous gambling activities [47]. Participants played on slot machines and had to decide whether to stop or continue after several rounds. Their behaviors were tracked and compared with their gambling goals, which were assessed through a questionnaire (eg, how much they wanted to win and how motivated they were to win).

### VR Therapies Are Generally Efficacious Compared to Other Treatments

The studies in this scoping review either examined the efficacy of VR alone or in comparison to traditional CBT. Comparing pre- and post-effects of VR therapy, VR therapy was found to be effective in reducing the severity of behavioral addictions [39,42-46]. For gaming disorders, the VR therapy group had significantly shorter gaming time (*P*=.04) and increased weekly physical activity values (*P*=.03) as compared to a control group that did not receive any treatment [47] and showed significantly reduced scores on the Young’s Internet Addiction Scale (YIAS; *P*<.01) after the treatment [43]. Practicing refusal skills during VR sessions also significantly lowered craving responses (*P*<.03) [44]. The Internet Gaming Disorder group had a higher frequency of halting the game after the risk/benefit assessment (*P*=.003) and anger expression task (*P*=.001) practices using VR as compared to baseline [45].

Similarly, for gambling disorders, VR therapy was effective in controlling gambling problems (*P*<.05) [39], and participants in the VR therapy group showed significant decreases across sessions in the subjective urge to gamble as compared to baseline (*P*=.04) [42]. However, one study found no reduction in urges to gamble after a single session of CET in a virtual gambling environment session [41]. It was speculated by the author that this could be due to the study having only one session of 20 minutes as compared to other studies with multiple sessions and longer durations. Overall, all but one study found promising results for the use of VR in the treatment of behavioral addictions.

When compared to other treatment modalities, VR therapy was found to be an effective and viable alternative [39,43,46]. Both CBT and VR therapy resulted in a reduction in the urge to gamble, with similar results regardless of the treatment modality (*P*<.001) [37]. The VR therapy program was shown to be as effective as the aerobic exercise program in reducing the severity of gaming disorder and anxiety, with both groups having significantly shorter gaming time (*P*=.04 and *P*=.04) and sedentary time (*P*<.001 and *P*=.03) [46]. Similarly, both CBT and VR therapy groups showed significant effects in reducing YIAS scores with no significant difference in YIAS score change between the CBT and VR therapy groups (*P*=.52) [43]. VR therapy was also effective when used in combination with CBT (*P*<.05) or relaxation exercises (*P*=.04) [39,42]. Furthermore, VR therapy demonstrated effectiveness in reducing the severity of online gaming addiction among individuals with attention deficit/hyperactive disorder symptoms by enhancing their engagement through greater immersion in the therapeutic process using VR. This reduced the challenge of maintaining their attention with conventional therapeutic techniques [43]. Two studies did not evaluate the efficacy of their treatments [40,47].

### Varied VR Treatment Programs With No Adverse Effects Reported

Across the 8 studies [39,41-47], with one [40] protocol study excluded, the treatment program varied in terms of type, frequency, duration, and the personnel who conducted the treatment (Table 1). In addition to investigating the use of VR therapy for the treatment of behavioral addictions, some studies [39,41,44,45] considered possible adverse effects, such as cybersickness, also known as simulator sickness. Cybersickness is a syndrome akin to motion sickness and is a potential side effect during and/or after exposure to various virtual reality environments. While motion sickness is triggered by actual movement, cybersickness is triggered by visual stimulation [50].

**Table 1:** Program details and cybersickness measurement.

Author (year of publication)	Exposure duration	Facilitated by	Measured cybersickness
Bouchard et al (2017) [39]	20 min	Therapists	Yes
Giroux et al (2013) [41]	20 min	Researcher	Yes
Lister et al (2016) [47]	5 min	Experimenter	No
Maden et al (2022) [46]	30 min	Physiotherapist	No
Park et al (2015) [42]	40 min, 5 separate scenes	Not stated	No
Park et al (2016) [43]	25 min	Psychiatrist	No
Shin et al (2018) [44]	Not stated	Not stated	Yes
Shin et al (2021) [45]	2 scenes, 10-15 minutes each	Therapist	Yes

In terms of duration, 5 studies had participants exposed to VR for at least 20 minutes [39, 41, 42, 43,46], while one study had participants in the VR environment for 5 minutes [47]. In one study, participants were exposed to 5 different scenes in 40 minutes [42]; however, the authors did not state if there were any breaks between the scenes. Similarly, a different study had participants in 2 different scenarios of about 10-15 minutes each [45], without indicating if a break was given in between. One study did not state the duration of virtual environment exposure [44]. Of the studies that specified treatment details, the number of treatments varied from one session [41] to 18 sessions [46].

Despite the varied frequency and duration of treatment, VR used in the treatment for behavioral addictions was not found to induce significant cybersickness. To measure the presence of cybersickness due to VR therapy, 3 studies used the cybersickness questionnaire [39,44,45], while one study used a post-VR exposure questionnaire [41]. The recorded levels of cybersickness did not increase in intensity compared to before VR therapy [39] or remained lower than established norms [44,45], affirming the feasibility of VR immersion for treating internet gaming disorder [44,45] and gambling disorder [39,41]. The other 5 studies [40,42,43,46,47] did not specify measuring cybersickness and did not report any negative side effects associated with VR therapy.

### Further Research Is Needed to Support the Use of VR in Behavioral Addiction Treatment

The findings from the included studies in this scoping review highlighted the potential for further exploration of VR in the treatment of behavioral addictions and suggested expanding the use of VR within and beyond the scope of behavioral addictions. All studies should report facilitators of the programs to ensure that adequately trained personnel can administer the intervention. Of the studies that reported this, VR therapy was facilitated by therapists (n=2), psychiatrists (n=1), physiotherapists (n=1), and the researchers themselves (n=2). Two articles did not state the facilitators. Additionally, 2 studies called for improving the efficacy of VR for treatment to address gambling addictions. Park et al [42] suggested investigating the optimal frequency and duration of VR sessions. Similarly, for gaming addictions, Shin et al [44] stated the need to focus on developing VR treatments for internet gaming disorders and comparing them with conventional treatment protocols.

Four studies suggested additional research to assess the effectiveness of combining VR with other types of therapy. Bouchard et al [39] suggested using VR with other forms of psychotherapy or mindfulness. Giroux et al [41] recommended combining VR treatment and relapse prevention with cognitive intervention for gambling problems. Shin et al [45] expressed interest in examining the long-term effects of skill learning in addressing gaming disorders when VR is combined with CBT. Giordano et al [40], whose study protocol aimed to compare combined VR therapy and CBT with traditional CBT, indicated a need for future studies to include a nontreatment control group to better analyze the efficacy of the combined intervention. Beyond behavioral addictions, Bouchard et al [39] recommended exploring the potential of VR applications in addressing other types of addiction. They also cautioned that there is a need for research on the potential of VR addiction [39].

Outside of using VR, Lister et al [47] suggested discovering if there is a better way to reduce problematic gambling outcomes (chasing and losing money), such as redefining goal-setting for gambling achievements. They suggested that setting realistic gambling goals might be more effective in reducing problem gambling compared to the current limit-setting tools such as limiting time or money. Park et al [43] and Maden et al [46] did not mention future research directions.

### Consultation Response

Consultation questions, along with an earlier draft of our manuscript, were sent to 6 stakeholders; however, only one responded after the initial email and 2 follow-up emails. Due to the low response rate, no formal analysis was conducted for this stage. The questions and responses are available in Multimedia Appendix 4. Our consultant (Dr. Bhing Leet Tan) has experience working as an occupational therapist and more than 11 years of experience as the head of the department at a mental health hospital. She has also published research on using VR for rehabilitation and AR for people with intellectual and developmental disabilities. She noted that while younger clients may find VR interventions more acceptable due to familiarity, older individuals, particularly those with gambling addictions, may require adjustment. She suggested grounding interventions within established frameworks like CBT or other frames of reference and providing interventions in multiple languages for psychoeducation. While she found it noteworthy that VR interventions showed positive outcomes in conflict resolution and assertiveness training, this did not come as a surprise to her due to VR’s prior effectiveness in social skills training. Nonetheless, she identified gaps in the literature, including specific VR hardware used and the sustainability of urge reduction gains over time.

## Discussion

### Principal Findings

This scoping review was conducted to provide an overview of existing literature on the use of AR and VR treatment for behavioral addictions. VR was found to be used for the treatment of problem video gaming and problem gambling. However, no AR studies were found. A breadth of studies from 4 countries and participant age ranges (children to older adults) were found; however, participants in the gaming studies were younger than participants in gambling studies. All 9 studies explored either problematic gaming or gambling, with study participants being mostly male, and ethnicity was rarely reported.

CET and skills training were the most common uses of VR in treatment to help with controlling urges and for role-playing to practice specific skill sets. Additionally, it was used as a platform to encourage a healthier lifestyle by introducing alternative activities. VR was applicable for both gaming and gambling disorders. Overall, VR therapy shows promise in the treatment of both gaming and gambling disorders, either as a standalone intervention or in combination with other treatments. It has also shown comparable results to other treatment modalities, showcasing its potential as a viable alternative to traditional treatment approaches. Nevertheless, these findings should be interpreted with caution, as the current body of evidence is still preliminary. A comprehensive systematic review assessing the methodological quality of existing studies is warranted.

Furthermore, most studies in this review tested for adverse effects, but none were identified. Overall, the results indicated that regardless of the treatment type, participants were able to spend up to 40 minutes in the VR environment without experiencing any adverse effects. Additionally, the varied facilitators did not seem to have an impact on cybersickness experienced by participants.

### Comparison to Prior Work

Compared to previous literature, this scoping review both supports and extends prior findings in several ways. Consistent with earlier reviews, this review confirms that most research on VR interventions for behavioral addictions is concentrated in a small number of countries [51], notably, Canada (33.3%) and South Korea (44.4%) for this study [28]. Furthermore, 75% of the articles on gaming disorder are from South Korea, possibly due to esports being a prominent culture in South Korea [52]. South Korea also has a very high proportion of gamers among the adolescent population (as high as 91.5%) [53]. This review identified an acute gap in research on the use of AR as an intervention for behavioral addictions, notwithstanding its emerging promise in other fields in mental health rehabilitation [54-56] and in substance use disorders [57,58].

While the practicality of VR in clinical use could be limited by financial constraints [28] and higher production costs [59], AR is an economical and accessible alternative with easy incorporation into smart phones and mobile devices without the need for additional equipment such as headsets for VR environments [60]. Additionally, the ecological validity is higher in AR, as the problematic behavior is embedded in the real environment where the user is allowed to use their own body to interact with virtual objects as opposed to participating via a virtual representation of their body in VR [23]. A possible explanation for the lack of AR treatment approaches could be due to a limited understanding of AR by health care professionals and a lack of awareness of its potential benefits. There may also be more ethical concerns when using AR since it incorporates real-world environments into treatment, potentially making cue induction too realistic with no gold-standard precautions in place to manage clients.

CET and skills training were the most common uses of VR in treatment. These showed mainly positive results, not only in being effective in reducing the symptoms and severity of the addictions but also being comparable to traditional treatment modalities such as CBT. This aligns with the results from studies of patients with panic disorder and agoraphobia where VR exposure therapy was found to be effective and equivalent to CBT alone [61]. In fact, a randomized controlled trial found that self-led VR-based CBT was also found to be effective for panic disorder [62]. Although many other studies using VR, most commonly in the form of CET, found decreased craving and dependence in people with substance use disorders [28], an earlier study found no significant change in craving after VR exposure [63]. Overall, the potential of the use of VR in the treatment of behavioral addictions is promising, especially in combination with CBT. Further research, however, is needed to establish best practices.

A majority of the studies in this review that tested for adverse effects used the Simulator Sickness Questionnaire to test for cybersickness, which is the most widely used measure of sickness in virtual environments [64]. It has been recognized that VR can cause discomfort and cybersickness after prolonged use [65]. However, no adverse side effects were reported in any of the identified studies. In 8 studies, the duration participants spent in the VR environment was within 20 minutes, aligning with studies that confirmed this duration of VR usage did not lead to adverse effects. One study indicated that the VR therapy was 40 minutes, spent in 5 separate scenes [42]. This longer duration also did not lead to adverse effects of using VR, specifically cybersickness. Therefore, in this scoping review, VR is deemed appropriate in the treatment of behavioral addictions, as no significant adverse effects have been reported.

Possible reasons for no reports of cybersickness may be that individuals with gaming and gambling disorders may be spending prolonged periods of time in front of screens, such that they have a higher tolerance to light movements in VR devices. Dużmańska et al [66] suggested that cybersickness can be avoided when the VR users are allowed enough time to adapt to the simulator conditions. Hence, prolonged periods of screen time during gaming and gambling can be a form of adaptation to the screen rate and locomotion, thus reducing the severity of cybersickness. Nevertheless, studies should screen for participants with known motion sickness, light sensitivities, epilepsy, and schizophrenia.

### Future Directions

Overall, the studies included in this review showed that VR was a viable mode of delivering therapy for behavioral addictions (gaming and gambling) and agree that further research is required to optimize the use of VR in treatment to maximize its potential. There is a scarcity of research on the topic of AR and VR in the treatment of behavioral addictions. Future programs that use AR or VR should consider publishing details of their program (eg, what their program was for and what tasks participants were asked to complete) and their effectiveness. Overall, more studies focusing on using AR as a treatment modality for behavioral addictions could be explored.

There is potential for AR and VR to be used in other types of problematic behaviors other than video gaming and gambling. Investigating cue exposure may provide a useful starting point, given that it is the most extensively studied intervention within the existing literature. As Bouchard et al [39] mentioned, combining VR with other psychotherapies is another avenue to explore in future intervention development. An 8-week mindfulness-based program was found to be effective for individuals with gambling problems [67]. A self-directed mindfulness program could be integrated with AR technology in the form of an app to facilitate increased accessibility to treatment. Comparison studies of VR treatment with pharmacological treatments could also be conducted. The ability to configure virtual environments also allows for personalization of treatment, potentially enhancing the effectiveness and receptivity of therapeutic interventions. All in all, VR treatments appear to be efficacious. However, long-term studies and evaluation of the quality of evidence are needed to determine its viability.

This review’s consultant points out that the potential of VR interventions among different age groups underscores its potential as a versatile therapeutic tool. She suggested the importance of integrating theoretical frameworks into VR interventions to enhance efficacy and address clients’ needs effectively. Further research is warranted to address the identified gaps and refine VR intervention strategies for optimal clinical outcomes.

### Strengths and Limitations

As this study was a scoping review, we did not aim to evaluate the risk of bias and quality of the studies found. First, only 9 articles from a small range of countries were found, which greatly limits the amount of evidence in evaluating the actual potential and efficacy of VR in behavioral addictions and in the different contexts. As more articles are published, a future systematic review may be warranted to synthesize the quality of the literature on AR and VR treatments in behavioral addictions. Second, there may be other relevant documents beyond the published articles and gray literature searches, not available in the public domain or published in other languages on this topic, which were not found by our research team and which could not be included in this review due to time and resource restrictions. To overcome this, requests have been made to the library to access full-text articles, and references of included studies were hand-searched for relevant studies. However, the diversity of studies in this review was still limited. Systematic searches of hospital, government, and global agencies may have revealed more studies.

Third, additional limitations based on this review’s findings are that only studies on the use of VR with gambling and gaming disorders were identified, despite the wide spectrum of problematic behaviors that are studied (eg, excessive social media use and online shopping). This limits conclusions that can be drawn about AR and VR for other types of behavioral harms relating to technology and internet use. Finally, there were different types of behavioral addictions and varying age groups and sexes of participants. Although it served the purpose of our review, the results may not be as straightforward in providing an accurate picture of the way AR and VR can be incorporated in effecting the treatment of behavioral addictions. On the other hand, this scoping review helps to consolidate available studies and provide an overview and potential directions that may enhance the use of AR and VR in behavioral addiction treatment.

### Conclusion

In this scoping review, we consolidated existing literature on the use of VR in the treatment of behavioral addictions, identifying types of behavioral addictions or harms that are being treated and the types of treatments or interventions that are conducted. This review shows that behavioral addictions in gaming and gambling can be effectively treated by using VR. For treatment, VR therapy can be used by itself or in conjunction with CBT for skills training and controlling urges. In research, VR can be used as a modality to simulate real events, such as to study gambling behaviors or the therapeutic effects of physical activities. The range of future research suggested by the studies shows that the full potential of VR therapy is yet to be realized. More research is needed to optimize VR treatment for behavioral addictions in terms of type, frequency, and duration. As further research progresses, this review hopes to encourage the continuous development of treatment options that advances with technology.

## Appendix

Multimedia Appendix 1 Search terms and keywords.

Multimedia Appendix 2 PRISMA-ScR checklist.

Multimedia Appendix 3 Summary of virtual reality applications in behavioural addictions.

Multimedia Appendix 4 Consultation questions and responses.

## Abbreviations

AR: augmented reality
CBT: cognitive behavioral therapy
CET: cue exposure therapy
ICD-11: International Classification of Diseases, 11th revision
JBI: Joanna Briggs Institute
PRISMA: Preferred Reporting Items for Systematic Reviews and Meta-Analyses
PRISMA-ScR: Preferred Reporting Items for Systematic Reviews and Meta-Analyses extension for Scoping Reviews
VR: virtual reality
WHO: World Health Organization
YIAS: Young’s Internet Addiction Scale

## Data Availability

All data generated or analyzed during this study are included in this published article and its supplementary information files.
