# Supplementary material for: Treating Behavioral Addictions With Augmented Reality and Virtual Reality: Scoping Review
Source: Interact J Med Res. 2026 Apr 30;15:e77011. doi: 10.2196/77011 (PMC13176813; doi:10.2196/77011)
Supplement: Multimedia Appendix 1 [file ijmr_v15i1e77011_app1.docx]

**Multimedia Appendix 1**

*Search terms and keywords*

| Term 1 AND | Term 2 AND | Term 3 |
| --- | --- | --- |
| "VR" OR "virtual reality" OR "AR" OR "augmented reality" | "therap*" OR "treatment*" OR "intervention*" OR "program*" | "internet addiction*" OR "behavio* disorder*" OR "behavio* addict*" OR "internet behavio* disorder" OR "internet behavio* issues" OR "online behavio* addiction" OR "internet behavio* addiction" OR "problematic internet use" OR "internet gaming disorder" OR "gaming disorder" OR "gaming addiction" OR "internet gaming addiction" OR "online gaming addiction" OR " gaming dependency" OR "internet gambling addiction" OR "online gambling disorder" OR "gambling disorder" OR "problem gambling" OR "gambling addiction" OR "online gambling addiction" OR "online shopping addiction" OR "online shopping disorder" OR "internet shopping disorder" OR "internet shopping addiction" OR “compulsive shopping” OR “impulsive buy*” OR "social media addiction" OR "social media disorder" OR "social media overuse" OR "internet overuse" OR "gaming overuse" OR "gambling overuse" OR "video gaming addict*" OR "video gaming overuse" OR "video gaming disorder" OR "problem video gaming" OR “compulsive buying” OR “buying disorder” |
