# Supplementary material for: Treating Behavioral Addictions With Augmented Reality and Virtual Reality: Scoping Review
Source: Interact J Med Res. 2026 Apr 30;15:e77011. doi: 10.2196/77011 (PMC13176813; doi:10.2196/77011)
Supplement: Multimedia Appendix 3 [file ijmr_v15i1e77011_app3.docx]

**Multimedia Appendix 3**

*Summary of virtual reality applications in behavioural addictions*

| Author, Year of publication, Location & Setting | Participants (number, age, sex & ethnicity) | Behavioural Addiction (type & assessment used) | Aim(s) of Study | Treatment Details | Outcome Measures | Results | Future Directions (as reported by authors) | Conclusion |
| --- | --- | --- | --- | --- | --- | --- | --- | --- |
| Bouchard et al., 2017, Canada; inpatient treatment centre  (Study 1) [39] | N = 64 Age range = 18-65;  Mean age not reported;  Sex not stated;  Ethnicity not stated  Control: N=36, occasional gamblers (play VLTs at most twice per year). | Potential gambling disorder in frequent gamblers who play video lottery terminals (VLTs) at least once a month, less severe than those diagnosed using the South Oaks Gambling Screen | To demonstrate that virtual reality (VR) immersions can trigger cravings in individuals with gambling disorder. | Participants played each of the following games for 7 mins: (a) Scrabble™ (control condition); (b) actual VLT; (c) a virtual bar with VLTs (At Fortunes); and (d) a virtual casino (3Dice).  One session, duration not stated. Facilitator not stated. | - After each game: Gambling Craving Scale (GCS) | - Statistically significant main effects on anticipation of fun and desire to gamble subscales of the GCS, but not on relief from negative affect. - Frequent gamblers experienced a significant increase in anticipation and desire to gamble with real VLTs or VR, compared to occasional gamblers. Urges to gamble strongly correlated with gambling addiction severity. | - Large-scale RCTs and research on VR addiction. - Examine sense of presence and environmental factors in VR immersions - Study the impact of virtual stimuli associated with other addictions - Combine VR with other forms of psychotherapy - Evaluate how VR can enhance the patient’s motivation during therapy | - VR gambling can trigger cravings in frequent gamblers. - Strong correlation between gambling urge intensity and gambling behaviour severity, suggesting that findings may be relevant to more severe gamblers. |
| Bouchard et al., 2017, Canada; inpatient treatment centre  (Study 2) [39] | N = 20  Median age (SD) = 45(12.6);  Sex = 35% female, 65% male;  Ethnicity not stated  Control: N = 14 | Pathological gambling per Diagnostic and Statistical Manual of Mental Disorders 4^th^ Edition (DSM-IV) | To document the potential benefits of two VR immersions in treating gambling disorder. | 28 days traditional cognitive behavioural therapy (CBT) program with two 20-minutes VR exposure sessions. Facilitated by therapists (bachelor's degree in psychology or social sciences).   - First VR session: identify situations that increase gambling risk and dysfunctional beliefs - Second VR session (last week of therapy): practice relapse prevention skills. - Both sessions: participants immersed in VR situations for 20 minutes.   Control group: 28-day CBT with two imaginal exposure exercises | - Before and after treatment: GCS - Before and after the first VR session: participants identified and listed personal high-risk thoughts and situations. - Before and after the second VR session: participants rated gambling desire intensity - Simulator Sickness Questionnaire (SSQ). | - Therapists were significantly more likely to ask patients to verbalise their thoughts and emotions in VR than in imaginal exercises,. - VR immersion significantly helped therapists identify more high-risk situations than imaginal exercises. - VR use did not lead to cybersickness symptoms.   Reduction in cravings was significant for both VR and control imagination conditions, with no differences between them. | Future directions as stated above | - Using VR in a clinical setting is beneficial when obtaining information about patients' thoughts, behaviours, and high-risk situations. - VR sessions were as effective as imaginal exposure exercises (can serve as a viable alternative during CBT for gambling disorder). |
| Bouchard et al., 2017, Canada ; inpatient treatment centre  (Study 3) [39] | N = 24;  Age range not stated;  Mean age (SD) = 47(12.8);  Sex = 50% female, 50% male;  Ethnicity not stated  Control: N = 11 | Gambling disorder per DSM-V | To assess the safety of an increased use of VR with CBT for gambling disorder, specifically in terms of the intensity of gambling cravings. | 28 days traditional CBT program with four 20- minute VR exposure sessions. Facilitated by therapists (bachelor’s degree in psychology or social sciences).  Sessions:   - 1^st^: participants identified high-risk situations. - 2^nd^ & 3rd: participants conducted cognitive restructuring. - 4th: practicing relapse prevention techniques.  Control group: immersed in a virtual environment with no gambling or money-related cues, and were encouraged to imagine being in a high-risk situation and to apply CBT techniques | - Before treatment: Canadian Problem Gambling Index, Diagnostic Interview for Gambling, Gambling Related Cognitions Scale - Immediately and 12, 24, 36-hours post-session: in-house questionnaire on intensity and frequency of gambling urges - 2 weeks after treatment: Canadian Problem Gambling Index, Diagnostic Interview for Gambling, Gambling Related Cognitions Scale | - The treatment was significantly effective in controlling gambling urges following the third VR session. VR does not elicit a stronger or longer-lasting urge to gamble compared to imaginal therapy, and the urge remains low. - The urge to gamble significantly declines over time in the first session), with significant drops in during the second and third sessions. The urge plateaus without reaching zero in the hours and days after the sessions, indicating the baseline level of desire. | Future directions as stated above | - No significant differences in lasting effects on urge to gamble between VR-induced and imagination-induced urges. - Immediate post-session urge to gamble is around 20%, for both conditions. - Four CBT sessions using VR for gambling disorder showed positive results with 50-56% success rates and medium effect sizes. |
| Giordano et al., 2022; Italy; hospital [40] | N = 60;  Age range= 18-65;  Mean age, sex, ethnicity not stated  Control: size not specified | Gambling disorder assessed by the Millon Clinical Multiaxial Inventory-III, Barratt Impulsiveness Rating Scale Italian version, and South Oaks Gambling Screen Italian version | To develop an innovative psychological treatment for gambling disorder using VR, and to verify its efficacy. | Eight CBT sessions and eight psychotherapy sessions based on VR CET, each with a different environment in *Alter Game*. Weekly, 1-hour sessions; facilitated by a therapist.   - 1st included virtual protocol and training. Systematic desensitization with diaphragmatic breathing was conducted. - 2^nd^ exposure to VR environments in this order: a psychologist’s office, a street with no game-related stimuli, a street with triggers, an empty tobacco shop, a full tobacco shop with sound stimuli, an empty slot room, a slot room with no audio-video stimuli, a slot room with active slot machines with audio-video stimuli, a slot room with active slot machines and alcohol and tobacco cues. Immersion proceeds for 20 mins.   Control group: 16 sessions of CBT | - Before treatment: Gambling Related Cognitions Scale Italian version, Multidimensional Gambling Self-Efficacy Scale, biofeedback parameters - During treatment: Visual Analogue Scale (VAS) for craving intensity, biofeedback parameters measurements during the entire exposure to the virtual environment. - After treatment: Independent Television Commission’s Sense of Presence Inventory | No results were provided as this was a protocol for a study. | Future studies could incorporate behavioural tasks and a non-treatment control group to better analyse efficacy of combined intervention. Future developments of *Alter Game* could include avatars and new virtual environments. | The authors expect that *Alter Game* could be used for relapse prevention in gambling disorder. It may also be useful for gamblers with a high comorbidity of gambling and substance use disorders and/or other addiction disorders. |
| Giroux et al., 2013;  Canada; university [41] | N = 10; Age range not stated;  Mean age (SD) = 63.4(7.2); Sex = 4 females and 6 males; Ethnicity not stated | Gambling addiction; Screened using the Canadian Problem Gaming Index and the Gambling Inventory | To explore the efficacy of a single session of cue-exposure to a virtual gambling environment using VR on the urge to gamble. | One session, 20 minutes, CET using a VR immersion apparatus to generate a virtual environment with gambling-associated stimuli. Facilitated by the researcher, the process consisted of four parts. 1. Increase participants’ initial urge to gamble. 2. Practice in the virtual environment to enable familiarisation. 3. Treatment: Participants were asked to follow an exposure sequence. This sequence was repeated for a total of five times. Intervals between each repetition was not stated. 4. Decrease participants’ urges by asking them to name five negative consequences of their gambling. | - Before session: urge to gamble and perceived self-efficacy. - During session: urge to gamble and perceived self-efficacy. - After session: urge to gamble, perceived self-efficacy, Post-exposure Questionnaire (motion sickness in VR, rating of similarity between the gambling environment and where they most often gamble, distress with regard to urge to gamble after exposure). | - Significant increase in the urge to gamble on VLTs in the practice environment and in the gambling environment. - No significant difference between the urge to gamble on VLTs in the practice environment and after exposure, or in the gambling environment and after exposure. | Explore combining two types of therapy for gambling problems, for example, CET and cognitive intervention for relapse prevention. | - Lack of significant difference between pre- and post- measures of urge to gamble and perceived self-efficacy during CET in a virtual gambling environment session. - 20-minute VRT session did not change the urge to gamble and perceived self-efficacy. |
| Lister et al., 2016; Canada, university [47] | N = 121; Age range = 18-40; Mean age = 19.8(2.8); Sex = 67 males and 54 females Ethnicity = majority identified as Caucasian (N = 68) | Problem gambling screened using Problem Gambling Severity index | To evaluate the effect of gambling goals on chasing behaviour beyond known factors (e.g., problem gambling severity, winning money motivations, approach/avoidance motivation). | One session, 5 minutes.  Participants played slot machines in a virtual casino, and were told they would have $20 to gamble where winning would be theirs. The virtual casino was programmed to fix participants experiencing nominal wins and losses. After the 30^th^ spin in five mins, a timer sounded, and participants were asked if they wished to continue.  The subsequent number of plays were noted as ‘chasing spins.’ All spins played after participants made their chasing decision were programmed as losses. | - Before treatment: gambling goals (e.g. winning money motivations; problem gambling severity; Behavioral Inhibition Scale; Behavioral Activation Scale; - During treatment: chasing decision: (yes/no after first 30 spins); betting behaviour (tracked by an experimenter). | - Most participants decided to chase spins for approximately 10 spins. - Higher gambling goals and greater problem gambling severity predicted relatively more chasing spins. - Being ‘motivated to win money’ did not affect the number of chasing spins. | Comparison should be made between 'responsible goal-setting tools' with current limit-setting tools. | Gambling goals were an important and novel factor for chasing spins in the face of loss (or wins), even after meeting winning money motivations. |
| Maden et al., 2022; Turkey; Physiotherapy Practice and Research Centre [46] | N = 44; Age range= 18-28; Mean age (SD) = 23.8(1.7) VRT; Mean age (SD) = 22.1(2.2) Aerobic training; Mean age (SD) = 22.2(1.4) Control; Sex = all males Ethnicity not stated  Control: N = 15 | Internet gaming disorder (IGD) screened using DSM-V; 11th Revision of the International Classification of Diseases (ICD-11) criteria for gaming disorder in the past 12 months | To compare the effects of VRT and aerobic training exercise programs on IGD severity, physical activity, physical fitness, and anxiety against the control group. | 18 sessions of 30 mins, 3 days a week over 6 weeks. Sessions were supervised by a physiotherapist. Both groups started with a 5-min warm up and ended with a 5-min cool-down on a treadmill.  VRT group: 15 participants played games using an Xbox Kinect 360 device. All games were played in standing in pairs which were randomly generated. The movement patterns of the games were boxing, hurdles, and beach volleyball.  Aerobic training group: 14 participants took part in training sessions consisting of loading  Control group: no treatment | - Before and after sessions: Gaming time ; The Internet Gaming Disorder Scale – Short Form (IGDS9-SF); Beck Anxiety Inventory; International Physical Activity Questionnaire - Short Form; Senior fitness Test; Predicted VO2 max; body mass index, height, and body weight; smoking status | - Post-training comparisons of gaming time and sedentary time were similar in VRT and aerobic training groups. - Compared to the control group, both experimental groups had significantly shorter gaming time and sedentary time; weekly physical activity values increased significantly; IGD severity reduced significantly - Both experimental groups also reported reduced anxiety levels | Not stated. | Both VRT and aerobic training was effective in treating IGD, with similar results in reducing IGD severity and anxiety. |
| Park et al., 2015; South Korea; outpatient clinic [42] | N = 12; Age range = not stated;  Mean age (SD) = 32.32(6.43);  Sex = all males;  Ethnicity not stated | Recreational gambling | To evaluate how five VR casino cues, used in CET, elicited subjective reactions and physiological responses.   To investigate the changes in participants' gambling urges following multiple sessions of repeated exposure and relaxation training. | 1 session was conducted first for feasibility and effectiveness, followed by five weekly 40-minute sessions. Facilitator not stated.  Participants watched a 3-min relaxation video, before being immersed in five casino scenes consisting of engaging in a casino setting, deciding on chip exchange amount, observing a jackpot scene, participating in a casino game and conversing about gambling with a colleague. | - Before, during and after session: Self-rating of urge using a scale of 0 (no desire) to 100 (intense desire), psychophysiological response data (electromyography, skin conductance, and heart rate) | - All scenes increased participants' self-rated urges, significantly. - After repeated exposure, the urge to gamble was reduced in two scenes: playing a casino game and discussing gambling with a colleague. - In playing a casino game, significant differences across sessions in subjective urge was elicited. - During the scene of discussing with a colleague, the level of the urges elicited fluctuated across sessions significantly. | - Investigate different cues for eliciting urges. - Determine optimal number of sessions to enhance therapeutic effects. | - The urge to gamble was strongest when exposed to a scene involving playing a casino game. - Repeated exposure significantly reduced gambling urges. - Combining VR CET with relaxation training is an effective approach for reducing gambling urges. |
| Park et al., 2016; South Korea; university hospital [43] | N = 36;  Age range not stated Mean age (SD) = 24.2(3.2) CBT group; Mean age (SD) = 23.6(2.7) VRT group; Mean age (SD) = 23.3(2.9) Control group; Sex = all males; Ethnicity not stated.  Control: N=12 casual gamers | Online gaming addiction was screened by a trained psychiatrist based on DSM-V. Evaluated through an interview and the 20-item Internet Addiction Test (IAT) | To study the therapeutic efficacy of VRT for online gaming addiction by balancing the limbic circuit via fMRI assessment before and after treatment.   To compare the efficacy of VRT for online gaming addiction compared to CBT. | CBT group: 8-session groups, 2hrs long, facilitated by experts (psychiatrist, nurse, psychologist & social worker). Each led a session of according to their expertise. The program was a total of 4 weeks with two sessions/week.  VRT group: A psychiatrist conducted a pre-interview (~30 mins) before the start of VRT sessions. Next, participants answered questions on what is precious to them and what is a problem caused by excessive gameplay. These were input into the VR program. Three steps were performed in the same order every session (totalling 25 mins): (a) relaxation to relieve tension by watching relaxing video with relaxing sounds (5 mins); (b) simulation of a high-risk situation to induce craving through exposure to gaming cues (10 mins); and (c) cognitive reconstruction through the use of aversion-inducing noise during exciting game scenes, together with aversive consequences of long-term pathological gameplay, followed by playing the recorded answers about the most precious things in their lives (10 mins)  Control group: no treatment | - Before treatment: resting-state fMRI assessment, IAT, Beck’s Depressions Inventory, Beck’s Anxiety Inventory, Korean version of the World Health Organisation adult attention deficit/hyperactivity disorder self-report scale - After treatment: resting-state fMRI assessment | Both CBT and VRT groups showed significantly reduced IAT scores with no significant difference in IAT score change between both groups. No significant difference in brain activity between both CBT and VRT groups. | Not stated. | - 8-sessions CBT and VRT are similarly effective at reducing severity of online gaming addiction. - VRT might aid in preventing habitual emotionless game use by facilitating limbic-regulated responses to rewarding stimuli. |
| Shin et al., 2018, South Korea; setting not reported [44] | N = 34 (IGD group);  N = 30 (control group);  Age range= 12-25;  Mean age (SD) (IGD group) = 17.2(4.57); Mean age (SD) (control group) = 18.6(4.98);  Sex = all males;  Ethnicity not stated  Control: N = 30 individuals without IGD | IGD diagnosis by psychiatrist interview according to the DSM-V | To explore the feasibility of VR application in IGD for adolescents and young adults, and to assess the effect of VR in cue reactivity. | In a single session, four tasks were conducted in VR: 1. At the Internet Cafe entrance, the counter clerk greets and encourages them to explore 2. Observation of conversation about game update/world championship 3. Gaming invitation to relieve exam stress  4. Gaming invitation to compete against opponents who criticized their game Participants started with task 1, followed by the other 3 tasks in different sequences. Duration not stated; facilitated by a researcher.  Control group: same treatment | - At the beginning: Assessment of demographic and clinical characteristics and IQ through Short form of the Wechsler Intelligence Scale for Children-Third Edition; Wechsler Adult Intelligence Scale-Revised; modified IAT. - At the entrance of a internet cafe VR environment and after each task: self-reported craving VAS - After virtual environment immersion: measures of the VR experience including Presence Questionnaire, SSQ. | - IGD group showed significantly higher craving scores and acceptance rate during gaming invitation than controls. - Both groups reported significantly higher cravings when entering the internet cafe and being invited to a game, compared to observing a conversation about internet games. Within the IGD group, game invitations elicited significantly more cravings than observing a conversation about internet games. - In both groups, craving responses to the refusal skills practice task were significantly lower than in the gaming invitation task and within the IGD group. - VAS cravings and modified IAT scores were positively correlated after entering the internet café. - Groups scored similarly on the PQ but differed on the SSQ. | Future research should focus on developing and comparing VR treatments with conventional treatment protocols. | - VR is effective in eliciting cue-induced craving in individuals - Practising refusal skills during VR sessions significantly reduces craving levels. - Higher levels of craving are induced when participants are invited to actively engage in gaming compared to merely observing a conversation about gaming. |
| Shin et al., 2021; South Korea; University [45] | N = 50; Age range not stated; Mean age (SD) (intervention group) = 21.78(2.33); Mean age (SD) (control group) = 21.65(1.83); Sex = all males Ethnicity of participants were Asian  Control:  N = 26 individuals without IGD | IGD screened using the DSM–V | To explore the potential use of a VR-based application to manage game-related conflicts with parents of young adults with IGD. | Two sets of VR tasks facilitated by a therapist: 1. Anger expression task. 2. Coping skill training task where participants used two coping skills a) risk/benefit assessment and b) perspective taking 10 to 15 mins per scenario.  Control group: same treatment | - Before treatment: Wechsler Adult Intelligence Scale-Revised; gaming frequency; - During treatment: gaming craving on a VAS; rating the usefulness of the strategy; modified version of the readiness to change questionnaire; extent of anger experience on VAS - After treatment: Presence Questionnaire; SSQ | - Participants experienced a high level of presence in the virtual environment, with a significant difference between IGD and control groups - IGD and control groups had similarly low levels of VR simulator sickness - IGD group reported significantly higher anger experience, and stronger urges to keep playing than the control group, but were more likely to stop the game after the risk/benefit assessment and after anger expression. - In the IGD group, fewer gaming hours correlated to perceiving coping skills as more useful than anger expression. - The control group saw more benefits than risks, and better understood their parents' perspective. In the IGD group, perceiving more benefits was linked to greater motivation to change gaming behaviour. | Combining VR with CBT to study long-term effects of learning through VR. | - VR program for IGD may be helpful for encouraging more desirable behaviours and managing gaming-related family conflict. - VR allows the opportunity to educate individuals with IGD about the progression of conflicts and to assess their dysfunctional thoughts behind the conflicts safely. |

*Note.* N = number of participants; VLT = video lottery terminal; VR = virtual reality; GCS = Gambling Craving Scale; RCT = randomised controlled trial; CET = cue exposure therapy; DSM-IV/V = Diagnostic and Statistical Manual of Mental Disorders 4th/5th Edition; CBT = cognitive behavioural therapy; SSQ = Simulator Sickness Questionnaire; VAS = Visual Analogue Scale; VRT = virtual reality therapy; IGD = Internet Gaming Disorder; ICD-11 = 11th Revision of the International Classification of Diseases; IGDS9-SF = The Internet Gaming Disorder Scale – Short Form; IAT = Young's Internet Addiction Test.

References:

39. Bouchard S, Robillard G, Giroux I, Jacques C, Loranger C, St-Pierre M, Chrétien M, Goulet A. Using virtual reality in the treatment of gambling disorder: the development of a new tool for cognitive behavior therapy. Front Psychiatry Switzerland; 2017;8:27. PMID:28286486

40. Giordano R, Donati MA, Zamboni L, Fusina F, Primi C, Lugoboni F. Alter game: a study protocol on a virtual “serious game” for relapse prevention in patients with gambling disorder. Front Psychiatry Switzerland; 2022;13:854088. PMID:35432033

41. Giroux I, Faucher-Gravel A, St-Hilaire A, Boudreault C, Jacques C, Bouchard S. Gambling exposure in virtual reality and modification of urge to gamble. Cyberpsychology Behav Soc Netw United States; 2013 Mar;16(3):224-231. PMID:23496679

42. Park C-B, Park SM, Gwak AR, Sohn BK, Lee J-Y, Jung HY, Choi S-W, Kim DJ, Choi J-S. The effect of repeated exposure to virtual gambling cues on the urge to gamble. Addict Behav 2015 Feb;41:61-64. PMID:25306387

43. Park SY, Kim SM, Roh S, Soh M-A, Lee SH, Kim H, Lee YS, Han DH. The effects of a virtual reality treatment program for online gaming addiction. Comput Methods Programs Biomed Ireland; 2016 Jun;129:99-108. PMID:26860055

44. Shin Y-B, Kim J-J, Kim M-K, Kyeong S, Jung YH, Eom H, Kim E. Development of an effective virtual environment in eliciting craving in adolescents and young adults with internet gaming disorder. PloS One United States; 2018;13(4):e0195677. PMID:29672530

45. Shin Y-B, Kim J-J, Kim H, Kim S-J, Eom H, Jung YH, Kim E. Managing game-related conflict with parents of young adults with Internet gaming disorder: development and feasibility study of a virtual reality app. JMIR Serious Games Canada; 2021 Jan 18;9(1):e22494. PMID:33459603

46. Maden C, Bayramlar K, Aricak OT, Yagli NV. Effects of virtual reality-based training and aerobic training on gaming disorder, physical activity, physical fitness, and anxiety: A randomized, controlled trial. Ment Health Phys Act. doi: 10.1016/j.mhpa.2022.100465

47. Lister JJ, Nower L, Wohl MJA. Gambling goals predict chasing behavior during slot machine play. Addict Behav 2016 Nov;62:129-134. doi: 10.1016/j.addbeh.2016.06.018
