# Supplementary material for: Treating Behavioral Addictions With Augmented Reality and Virtual Reality: Scoping Review
Source: Interact J Med Res. 2026 Apr 30;15:e77011. doi: 10.2196/77011 (PMC13176813; doi:10.2196/77011)
Supplement: Multimedia Appendix 4 [file ijmr_v15i1e77011_app4.docx]

**Multimedia Appendix 4**

Consultation Questions and Responses

|  | Question | Response |
| --- | --- | --- |
| 1 | Would implementing this be realistic in your practice? | The different VR interventions use varying psychotherapeutic strategies, some with theoretical basis, some without. In many of these interventions, participants reported increased cravings while being immersed in the VR scenarios. Hence, there is a need to be clear of the objectives of the VR interventions. Some of them are used to induce addictions urge, so that participants can practise refusal skills or other CBT self-talk strategies. If used within a clear intervention frame of reference, some of these interventions can be useful. |
| 2 | What might your clients think of this? | Young clients are familiar with VR, so there should be some acceptability. Older persons with gambling addictions may take some time to get used to, but the immersive experience may provide a context that clients can quickly relate to and therapist can use the opportunity to work on self-management of gambling urges etc. |
| 3 | What would you do the same or differently from what has been done? | Many of these interventions are not grounded in a particular treatment frame of reference. It will be useful to deliver the intervention in the context of CBT, motivational interviewing or solution-focused approach. If it is used for psychoeducation purpose[s], a variety of languages will be helpful. |
| 4 | Were any of the findings surprising to you? | It is interesting to read that some of these VR interventions have reported positive findings in conflict resolution, assertiveness skills etc. This is certainly one area that VR can be used for skills training purpose. |
| 5 | Do you agree/disagree with the findings? | VR has been used successfully in social skills training, hence this is not surprising. |
| 6 | What do you think is missing from the literature? | It is assumed that all these studies used VR on a head mounted display? [Did] any study use CAVE? VR classes? For studies that showed [a] reduction of urges, were the gains sustained over a period of time? |

Note: Square brackets were used to denote corrections in grammar.

Note: VR = virtual reality; CBT = cognitive behavioural therapy; CAVE = cave automatic virtual environment.
